# High-throughput sequencing of *Medicago truncatula *short RNAs identifies eight new miRNA families

**DOI:** 10.1186/1471-2164-9-593

**Published:** 2008-12-09

**Authors:** Gyorgy Szittya, Simon Moxon, Dulce M Santos, Runchun Jing, Manuel PS Fevereiro, Vincent Moulton, Tamas Dalmay

**Affiliations:** 1 School of Biological Sciences, University of East Anglia, Norwich, NR4 7TJ, UK; 2 School of Computing Science, University of East Anglia, Norwich, NR4 7TJ, UK; 3 Laboratory of Plant Cell Biotechnology, ITQB/IBET – Apt 127, 2781-901 Oeiras, Portugal

## Abstract

**Background:**

High-throughput sequencing technology is capable to identify novel short RNAs in plant species. We used Solexa sequencing to find new microRNAs in one of the model legume species, barrel medic (*Medicago truncatula*).

**Results:**

3,948,871 reads were obtained from two separate short RNA libraries generated from total RNA extracted from *M. truncatula *leaves, representing 1,563,959 distinct sequences. 2,168,937 reads were mapped to the available *M. truncatula *genome corresponding to 619,175 distinct sequences. 174,504 reads representing 25 conserved miRNA families showed perfect matches to known miRNAs. We also identified 26 novel miRNA candidates that were potentially generated from 32 loci. Nine of these loci produced eight distinct sequences, for which the miRNA* sequences were also sequenced. These sequences were not described in other plant species and accumulation of these eight novel miRNAs was confirmed by Northern blot analysis. Potential target genes were predicted for most conserved and novel miRNAs.

**Conclusion:**

Deep sequencing of short RNAs from *M. truncatula *leaves identified eight new miRNAs indicating that specific miRNAs exist in legume species.

## Background

Gene expression is regulated at several layers in plants to ensure optimal temporal and spatial accumulation of proteins. One of the latest discovered regulatory layers involves short RNA (sRNA) molecules 21–24 nucleotides in length that act post-transcriptionally [1]. There are surprisingly many different sRNAs in plant cells indicating an extensive role for these molecules [2]. Plant sRNAs are produced from double stranded RNA (dsRNA) by one of the four Dicer-like proteins (DCL1-4). The different DCL proteins process dsRNAs generated by diverse pathways [1]. MicroRNAs (miRNAs) are produced from partially complementary dsRNA precursor molecules (pre-miRNA) [3]. Pre-miRNAs are originally single stranded RNAs with hairpin structures and recognized by DCL1 [4]. The other large class of plant sRNAs is small interfering RNAs (siRNAs). siRNAs are processed from dsRNAs usually generated by one of the RNA Dependent RNA Polymerases (RDRs). Trans-acting siRNA (ta-siRNAs) precursors are generated by RDR6 [5,6] and heterochromatin siRNA precursors are made by RDR2 [7]. Another group of siRNAs, the natural antisense siRNAs (nat-siRNAs), are processed from dsRNA produced by overlapping antisense mRNAs [8].

MiRNAs are the best characterized sRNAs in plants [9]. The primary transcript (pri-miRNA) is transcribed by RNA polymerase II and contains an imperfect hairpin structure. DCL1 trims this hairpin structure producing the pre-miRNA and then a second cleavage by DCL1 produces the miRNA/miRNA* duplex [10]. This molecule has a two nucleotide 3' overhang at each side of the duplex and contains a few mismatches [9]. One of the strands of the miRNA/miRNA* duplex is integrated into RISC (RNA induced silencing complex). This strand is called mature miRNA and the partially complementary miRNA* strand gets degraded, although in most cases the miRNA* strand also accumulates at a lower level [9]. RISC finds specific mRNAs because the incorporated mature miRNA can anneal to partially complementary target sites [3]. Target sites show near perfect matches to plant miRNA sequences and initially it was thought that all target mRNAs are cleaved by RISC [3]. Recently it was shown that the translation of plant mRNAs is also suppressed without a cleavage [11].

Most plant miRNA families have been identified by traditional Sanger sequencing method in model species with known genome sequences (Arabidopsis, rice and poplar) and most miRNAs are conserved across plant families [12]. However, some miRNAs are species/family specific and Allen et al. [13] suggested that these "young" miRNAs have evolved recently, in contrary to the conserved miRNAs ("old" miRNAs). Since non-conserved miRNAs are often accumulated at a lower level than conserved miRNAs, traditional small-scale sequencing primarily reveals conserved miRNAs. Establishment of high-throughput technologies has allowed the identification of several non-conserved or lowly expressed miRNAs through deep sequencing, e.g. in Arabidopsis, wheat and tomato [14-17]. Here we describe the deep sequencing of short RNAs extracted from *M. truncatula *leaves and the experimental validation of eight novel miRNAs.

## Results

### Deep sequencing of *M. truncatula *short RNAs

Two separate cDNA libraries of short RNAs were generated from *Medicago truncatula *leaves and both libraries were sequenced by Solexa (Illumina). The first PCR product was quantified by nanodrop and 5 pM was loaded to Solexa, which yielded 5942 clusters and 872,048 sequence reads. The second sample was also quantified on polyacrylamide gel because the capacity of Solexa is higher than this. Since the nanodrop gave an approximately 50 times higher concentration than quantification on a gel we concluded that the nanodrop overestimates the concentration of the PCR product. To obtain more sequences, we loaded four times of the amount that we would have loaded based only on the nanodrop reading and this yielded 23301 clusters and 3,076,823 sequence reads. It is worth mentioning that loading based on only quantification on gel would have led to overloading the Solexa and would have produced many but unreliable sequences.

**Figure 1:**
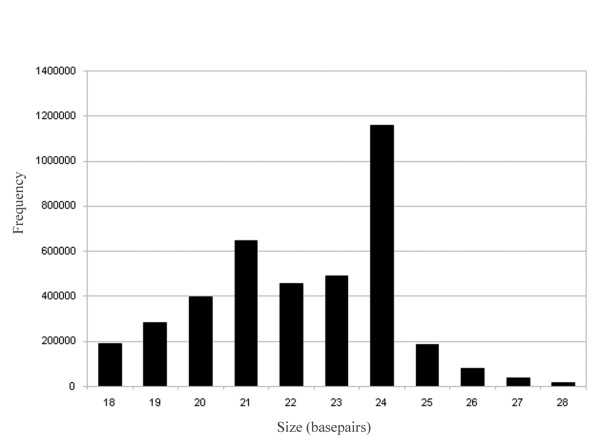
Size distribution of sequenced short RNAs.

The two sets of reads were combined and analysed together (Table 1) using the miRCat pipeline we have developed earlier [18]. The size distribution of sequence reads showed that the 24 nt class was the most abundant group of sRNAs followed by the 21 nt sequences and then the 23, 22 and 20 nt reads (Figure 1). The almost four million reads represented 1,563,959 distinct sequences suggesting that the library is still not saturated. Out of the four million reads 2,168,937 matched to the genome without any mismatches, representing 619,175 sequences.

**Table 1:** Distribution of short RNAs

	Number of reads	Number of distinct sequences
Total	3948871	1563959

Mapping to the genome without a mismatch	2168937	619175

Known miRNAs (exact match)	174504	25

New miRNA candidates With sequenced miRNA*	3370	8

New miRNA candidates With sequenced miRNA*	1272	18

Number of reads and distinct sequences are shown of different categories of sequences.

### Conserved miRNAs

First we looked for known miRNAs by comparing our library to known miRNAs from other plant species. 174,504 reads corresponding to 25 conserved miRNA families showed perfect matches to known miRNAs. We analysed the number of reads for conserved miRNAs and miR156, 159 and 166 were represented most frequently in the library (Table 2). Allowing one or two mismatches between sequences in our library and sequences in miRBase increased the number of conserved miRNA families in *M. truncatula *to 31 (Table 2). Next we predicted target genes and putative targets were identified for 27 out of 31 conserved families (Additional File 1). No targets were found in *M. truncatula *for miR163, miR169, miR394 and miR894. We found homologs of known miRNA target genes for several conserved *M. truncatula *miRNAs, such as SBP for miR156, NAC for miR160, AGO1 for miR168, bZIP for miR165, GRAS for miR171, AP2 for miR172 and low affinity sulphur transporter for miR395. On the other hand we predicted many genes with unknown function and hypothetical genes for miRNA targeting (Additional File 1) and careful analysis of these potential targets will contribute to our understanding of the role of miRNAs in legume plants.

**Table 2:** Conserved miRNAs

**miR**	**Exact**	**Shorter**	**Longer**	**1 mismatch**	**2 mismatches**	**Total**
miR156	37580	5944	1112	14906	11423	**70965**

miR157	0	0	0	0	206	**206**

miR158	14	1	3	3	1	**22**

miR159	32003	30842	5825	1887	2454	**73011**

miR160	89	35	19	7	7	**157**

miR161	2	0	0	0	0	**2**

miR162	49	4	7	9	3	**72**

miR163	6	4	0	0	0	**10**

miR164	11815	794	210	639	213	**13671**

miR165	256	129	49	56	59	**549**

miR166	56402	12792	6349	1276	8253	**85072**

miR167	2729	623	420	114	133	**4019**

miR168	4404	429	173	338	159	**5503**

miR169	83	39	173	13	25	**333**

miR171	26	30	5	21	30	**112**

miR172	195	53	91	39	29	**407**

miR173	6	2	0	0	0	**8**

miR319	27322	944	3414	1075	1076	**33831**

miR390	729	154	32	55	43	**1013**

miR393	20	2	1	2	2	**27**

miR394	0	1	70	0	25	**96**

miR395	0	42	0	0	34	**76**

miR396	453	1279	158	41	466	**2397**

miR397	0	1	0	0	0	**1**

miR398	20	15	3	2	1	**41**

miR399	2	2	0	0	0	**4**

miR400	1	0	0	0	0	**1**

miR403	0	1	0	0	0	**1**

miR408	293	22	10	35	25	**385**

miR828	0	0	1	1	0	**2**

miR894	5	1	103	2	38	**149**

**Total**	**174504**	**54185**	**18228**	**20521**	**24705**	**292143**

Number of reads are shown for sequences that are similar to conserved miRNAs identified in other species. Figures are shown separately for sequences with exact matches and length, exact matches but shorter or longer than known miRNAs and for sequences with one or two mismatches to known miRNAs.

### Novel miRNAs

**Figure 2:**
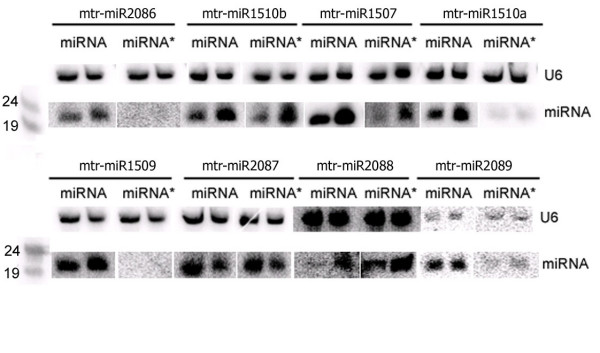
**Expression of new miRNAs**. Total RNA from stems and leaves of *M. truncatula *was analysed by Northern blot. The numbers correspond to ID numbers in Table 3. Size markers (19 and 24 nt RNA oligonucleotides) are shown on the left. The membranes were also hybridised with a probe specific to U6 [17].

The flanking regions of the 2,168,937 sequence reads matching the genome were subjected to secondary structure analysis. We used the strict criteria suggested by Jones-Rhoades et al. [9] to identify potential miRNA loci. 32 short RNA producing loci corresponding to 26 sequence reads could be folded into step-loop structures (Additional File 2). Nine of these loci produced eight distinct sequences, for which we also sequenced the miRNA* sequences (Table 3). These eight sequences were considered as novel legume specific miRNAs based on Rajagopalan et al. [15], who demonstrated that loci producing sequenced mature miRNAs and miRNA* sequences are miRNA loci. The other 23 loci producing 18 sRNAs were considered as putative new miRNA genes since at the moment there is not sufficient evidence to classify them as miRNA genes. Accumulation of all eight new miRNAs and most miRNA*s were validated by Northern blot analysis (Figure 2).

**Table 3:** Potential new miRNA genes

sRNA	Chr	Start	No reads	Sequence	N. blot	miRNA*
miR2086	MtChr2	22878303	1164	GACATGAATGCAGAACTGGAA	+	Yes

C2	MtChr5	23546032	485	TGAGAATGTTAGATACGGAAC	-	No

miR1510b	MtChr5	17425793	721	ACATGGTCGGTATCCCTGGAA	+	Yes

miR1507	MtChr8	34001142	1170	CCTCGTTCCATACATCATCTAG	+	Yes

C5_1	MtChr4	10481611	414	GGTCCCCGGCAGCGGCGCCA	-	No

C5_2	MtChr4	10570962	414	GGTCCCCGGCAGCGGCGCCA	-	No

C5_3	MtChr4	10515784	414	GGTCCCCGGCAGCGGCGCCA	-	Yes

miR1510a	MtChr5	17429726	170	CGGAGGATTAGGTAAAACAAC	+	No

C7	MtChr2	13077141	50	AAAAGAATACTCATACATAACATT	+	No

C8	MtChr7	2887662	29	TGTAAGGGATGATGCAAATGAGAG	+	No

C9_1	MtChr2	6722889	28	AGGGTGCTTAGATATAGGGACGGA	+	No

C9_2	MtChr8	2492462	28	AGGGTGCTTAGATATAGGGACGGA	+	No

C9_3	MtChr8	10174192	28	AGGGTGCTTAGATATAGGGACGGA	+	No

miR1509	MtChr7	31515912	90	TTAATCTAGGAAAATACGGTG	+	Yes

C12	MtChr2	26140680	19	GAAAGGACTAAAACCAGGAACGGA	-	No

C13	MtChr2	25488088	24	TACGCAGGAGAGATGATGCCG	-	No

C14	MtChr5	28947435	44	CTTGGACTTCAAGAAACAAC	-	No

miR2087	MtChr2	9824368	35	GAAGTAAAGAACCGGCTGCAG	+	Yes

C16	MtChr1	29344468	38	TAACTGCCGAGGCGTGCTGA	-	No

C17	MtChr4	20677608	30	TGATGACGGAAGAAATCCAAA	-	No

C18	MtChr6	2189030	16	AGATGGAGATTAGACGGCTCA	-	No

C19	MtChr5	13285282	16	AGATTTAGGCGGCTTTGAACT	-	No

C20	MtChr6	8477357	22	TGGACTGAAGACAAAGAGAAA	-	No

C21_1	MtChr4	33246760	13	ATTTTTCAGCGGAAGTTAA	-	No

C21_2	MtChr4	27138541	13	ATTTTTCAGCGGAAGTTAA	-	No

miR2088_1	MtChr8	14579972	11	AGGCCTAGATTACATTGGAC	+	Yes

miR2088_2	MtChr8	14675564	11	AGGCCTAGATTACATTGGAC	+	Yes

C23	MtChr5	31612014	12	TGTTGGAACACGGCTTAAGGT	-	No

C24	MtChr3	13221230	12	TAGGTTTGAGAAAATGGGCA	-	No

C25	MtChr8	24335508	11	TCTGTCAGTAGACTCAAT	-	No

C26	MtChr5	28244439	9	AAGGGCATATGTTAAGGAAC	-	No

miR2089	MtChr5	17442776	9	TTACCTATTCCACCAATTCCAT	+	Yes

The first column (sRNA) shows the ID number of the sequences. The second and third columns show the genomic location of the putative miRNAs (Chr: chromosome; Start: starting position on the chromosome). The number of reads are indicated in the fourth column and the sequences themselves are listed in the fifth column. The results of the Northern blot analysis are shown in the sixth column (+: detected; -: not detected). Whether the miRNA* sequence was sequenced or not is indicated in the last column.

We also predicted target genes for putative miRNAs (Additional file 3) and the new miRNAs with sequenced miRNA* (Additional file 4). Five out of the eight new miRNAs have potential targets in the available *M. truncatula *genome. It is interesting that two of these five new miRNAs potentially target disease resistance genes suggesting a role for miRNAs in the regulation of biotic stress response.

## Discussion

### Deep sequencing of *M. truncatula *short RNAs

Most conserved plant miRNAs were identified by traditional Sanger sequencing. Due to the relatively small number of sequences generated this method primarily discovered conserved miRNAs. Recent progress in sequencing technologies allows deep sequencing of large libraries of short RNAs [2,7,17,19-23]. The longest reads are obtained by the 454 technology, which currently gives reads of 250–300 base pairs (bp). However, this technology yields much less reads than other techniques (about 400 000 per sample) [17]. The Solexa platform (Illumina) generates shorter reads (up to 35 bp) but yields 1–3 million reads per sample [22]. The other very high-throughput technique, massively parallel sequencing (MPSS), gives more reads than Solexa but the reads are even shorter, only 17 bp [23]. miRNAs are only 21–23 nt sequences therefore even MPSS can identify them, although this technology generates shorter than 21 bp reads. Because of this it is not the ideal choice for miRNA discovery but it is useful for profiling conserved miRNAs. We decided to use the Solexa platform to test whether new miRNAs can be found in the model legume *M. truncatula*.

We found the quantification of PCR product problematic because the nanodrop overestimated the concentration and using quantitative markers on polyacrylamide gel seemed to underestimate the concentration. The difference between the two approaches was 50 fold. Loading 5 pM sample according to the nanodrop result yielded relatively low number of sequences (872,048 reads), well below the capacity of Solexa. However, loading 50 times more would have given poor quality sequences due to overloading. We decided to load four times of the amount suggested by the nanodrop and this approach yielded sufficient number (more than 3 million) and good quality reads. Many short sequences did not map to the genome but it is not known what the explanation is for that. One possibility is that the rate of sequencing error is high and many short reads contains mistakes. The other possibility is that there are polymorphisms between the species we used and the one that was used for the genome sequencing. It is also possible that plants used in these experiments were infected symptomless pathogens and genomes of those organisms contaminated our libraries.

First we searched for conserved miRNAs in the combined, almost four million reads from the two separate sRNA libraries. MiRNAs showing perfect matches to 25 conserved families were found in our combined library, which is more than was identified using bioinformatics approaches [24-26]. Allowing up to two mismatches further increased this number to 31 families. This work experimentally verified all conserved miRNAs predicted by [[24,25] and [26]]. However, none of the predicted new miRNAs [26] were found and the eight new miRNAs we found were not predicted by any of the three studies [[24,25] and [26]] illustrating the benefit of the sequencing approach. Target prediction for conserved miRNAs found several genes showing homology to genes validated in other species. However, several predicted targets are new without known function indicating that more work is required to elucidate the role of miRNAs in legumes.

### New miRNAs

Validation of non-conserved miRNAs can be achieved through several ways. One possibility is providing evidence of biogenesis characteristic of miRNAs. This involves the identification of stem-loop structure of the potential pre-miRNAs and sequencing of the miRNA* molecules [9]. In the absence of evidence for biogenesis, functional data can complement the predictions based on structural analysis. MiRNA mediated cleavage is located in a specific site within the target site and this can be identified by 5'RACE analysis. We have found eight new non-conserved miRNAs supported by biogenesis data since the miRNA* strands were sequenced and detected by Northern blot analysis. We also found 18 other putative miRNAs based on structural data. However, we did not sequence the miRNA* strands for these and we do not have functional proof therefore at the moment these are only putative miRNAs.

The role of miRNAs in development and abiotic stress response is well documented. Several transcription factors involved in development are targeted by miRNAs [27]. Other miRNAs play a role in nutrient assimilation and responses to drought, cold and other abiotic stresses [1,28]. Reports of miRNAs involved in biotic stress response are less common. Navarro et al [29] showed that bacterial flagellin expression induced miR393 and F-box auxin receptor genes were regulated by miR393. Two of the new miRNAs are predicted to target disease resistance genes suggesting a more extensive role for miRNAs in biotic stress response. Targets of the other new miRNAs also include beta-glucan-binding protein, peptidyl-prolyl cis-trans isomerise and a hypothetical protein. The biological importance of the potential regulation of these genes by miRNAs needs further investigation.

## Conclusion

High-throughput sequencing analysis of sRNAs from *M. truncatula *leaves identified eight new miRNAs, which were not found in other species. Sequencing sRNAs from specific tissues of legume plants by deep sequencing is expected to reveal more new miRNAs.

## Methods

### Cloning of *M. truncatula *sRNAs and Northern blot analysis

RNA was extracted using the miRVana kit (Ambion) from the aerial part (including stems and leaves) of nine week old *Medicago truncatula *Gaertn. cv. Jemalong seedlings. Plants were grown in pots with soil in a growth chamber having a photoperiod of 16/8 h day/night, a thermoperiod of 25/18°C day/night and humidity of 40%. Small RNA fraction between 19–24 nt was isolated from 15% denaturing polyacrylamide gel and 15 μg was ligated to adaptors without de-phosphorylating and re-phosphorylating [30]. The short RNAs were converted to DNA by RT-PCR and the DNA was sequenced on a Solexa machine (Illumina). 15 μg of RNA extracted from *M. truncatula *leaves was analysed by Northern blot as described by Pall et al. [31].

### Sequence analysis

The *Medicago truncatula *genome sequence (version 2.0) was downloaded from the Medicago Sequencing Resource website  and sequence reads were mapped to the genome using PatMaN [32]. miRNA candidates were generated by miRCat (; [33]) using default parameters. DFCI Medicago Gene Index v 9.0 was used for the target predictions: . Target searches were performed on the srna-tools website: [33].

The sequences can be found in GEO under GPL7704 platform (High-throughput sequencing of small RNAs in *Medicago truncatula*), series GSE13761, samples GSM346592 and GSM346593.

## Authors' contributions

SG cloned the short RNAs and carried out most of the Northern blot analysis; SM analysed the sequences and was supervised by VM; DMS grew the plants and prepared the RNA; RJ carried out some of the Northern blot analysis; MPSF and TD designed and coordinated the study; TD analysed the results and wrote the manuscript.

## Supplementary Material

Additional file 1 **Predicted targets of conserved *M. truncatula *miRNAs.** The data provided describes the predicted target genes of conserved miRNAs found in M. truncatula. Click here for file

Additional file 2 **Predicted secondary structures of new and putative *M. truncatula*.** The data shows the predicted secondary structures of validated and putative new *M. truncatula *miRNAs. Click here for file

Additional file 3 **Predicted targets of putative miRNAs.** This table provides a list of predicted target genes of potential but not validated *M. truncatula *miRNAs. Click here for file

Additional file 4 **Predicted targets of new validated *M. truncatula *miRNAs.** This table provides a list of predicted target genes of new *M. truncatula *miRNAs with sequenced miRNA*. Click here for file
