# Supplementary material for: High-throughput sequencing of Medicago truncatula short RNAs identifies eight new miRNA families
Source: BMC Genomics. 2008 Dec 9;9:593. doi: 10.1186/1471-2164-9-593 (PMC2621214; doi:10.1186/1471-2164-9-593)
Supplement: Additional file 2 — Predicted secondary structures of new and putative M. truncatula. The data shows the predicted secondary structures of validated and putative new M. truncatula miRNAs. [file 1471-2164-9-593-S2.pdf]

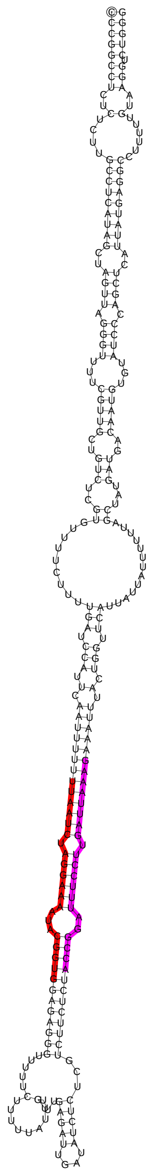

Secondary structure for 'C10'

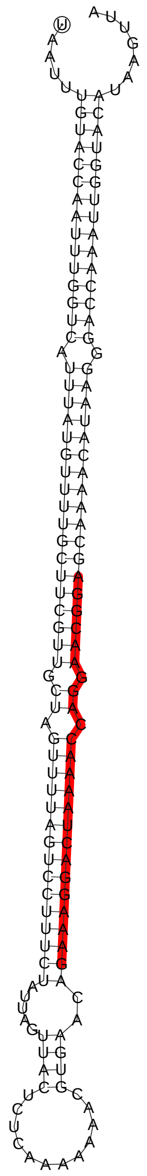

Secondary structure for 'C12'

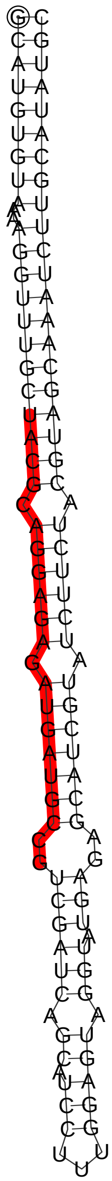

Secondary structure for 'C13'

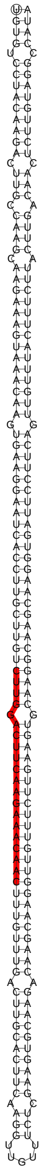

Secondary structure for 'C14'

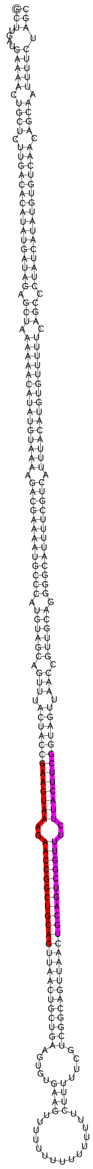

Secondary structure for ’C15’

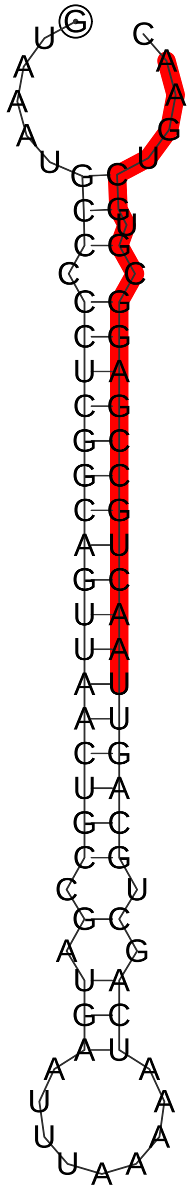

Secondary structure for 'C16'

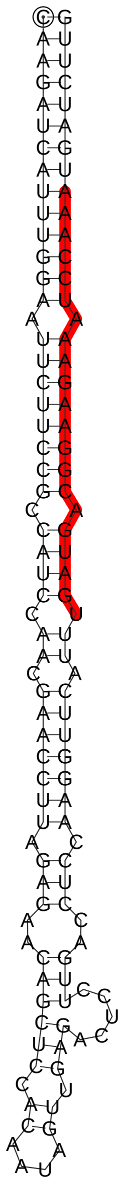

Secondary structure for ’C17’

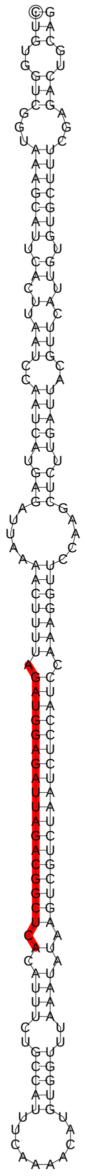

Secondary structure for ’C18’

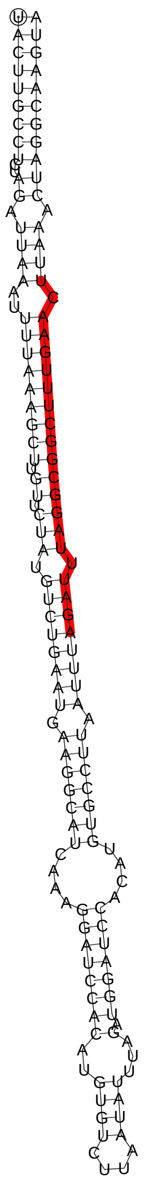

Secondary structure for 'C19'

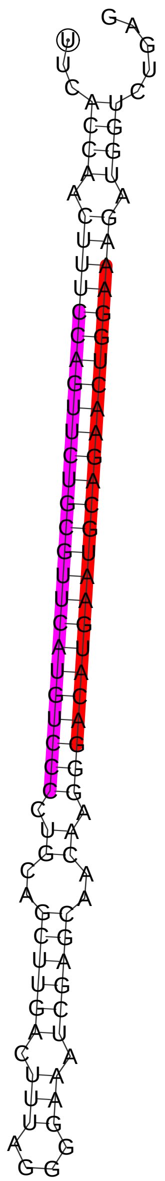

Secondary structure for 'C1'

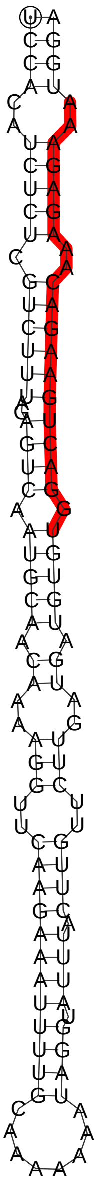

Secondary structure for ’C20’

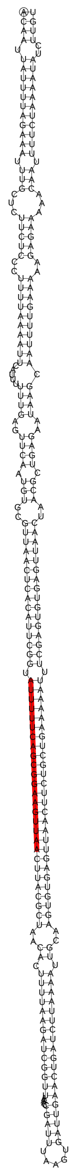

Secondary structure for ’C21_1’

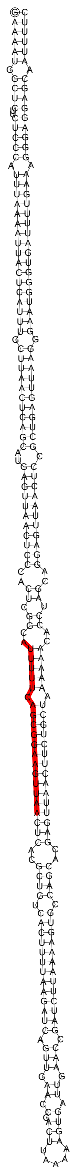

Secondary structure for ’C21_2’

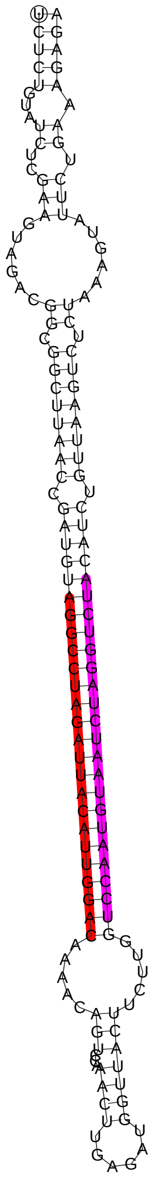

Secondary structure for ’C22’

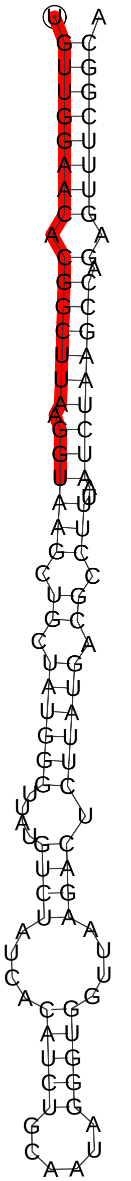

Secondary structure for ’C23’

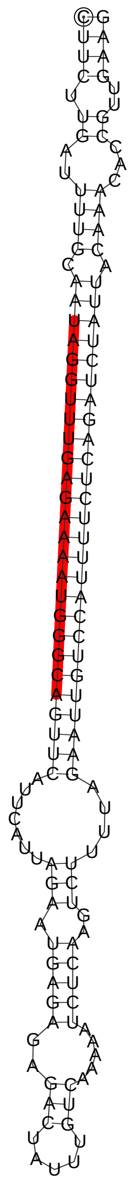

Secondary structure for ’C24’

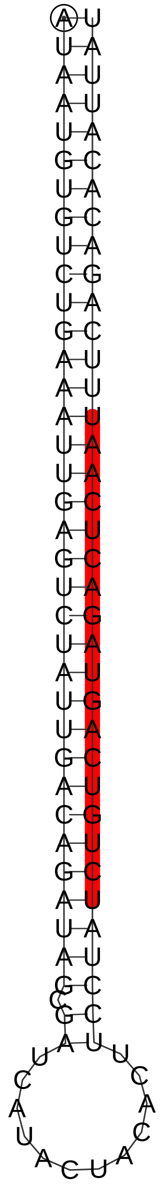

Secondary structure for ’C25’

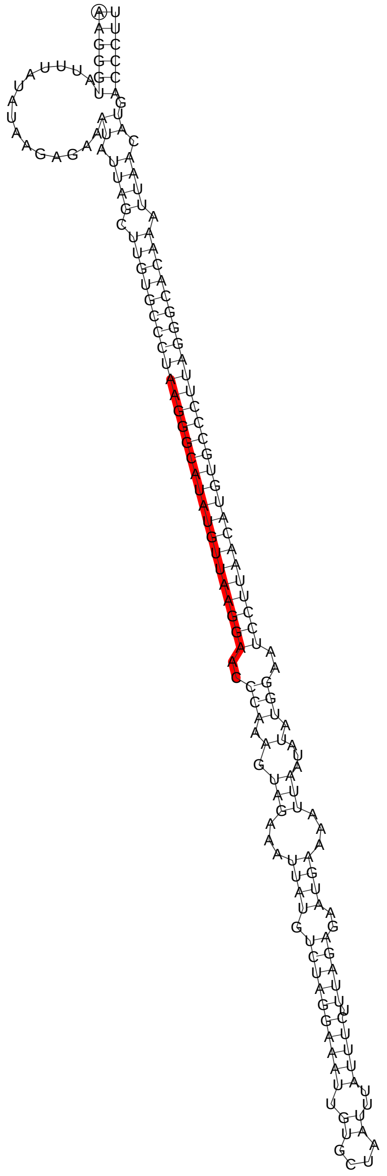

Secondary structure for ’C26’

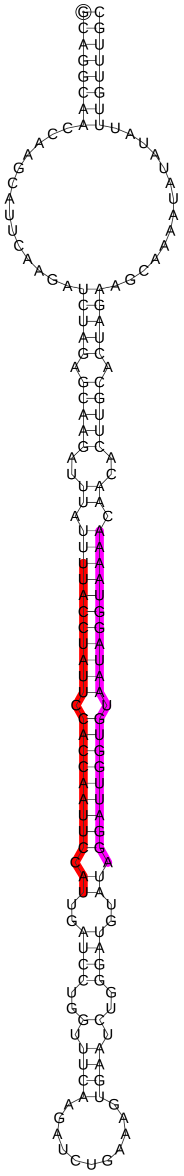

Secondary structure for ’C27’

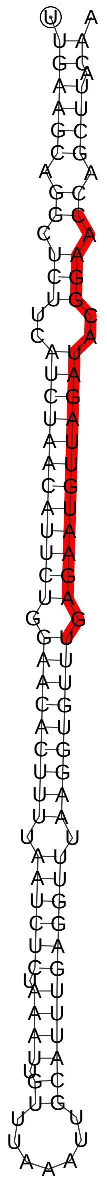

Secondary structure for ’C2’

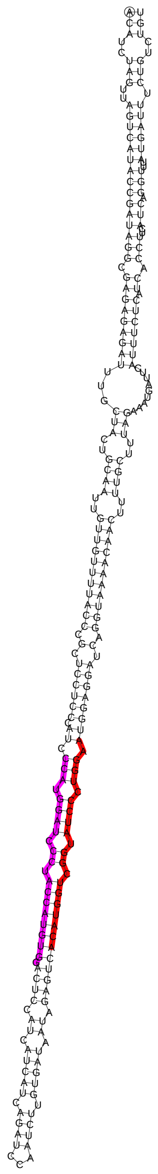

Secondary structure for ’C3’

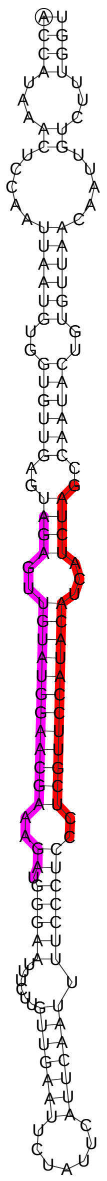

Secondary structure for ’C4’

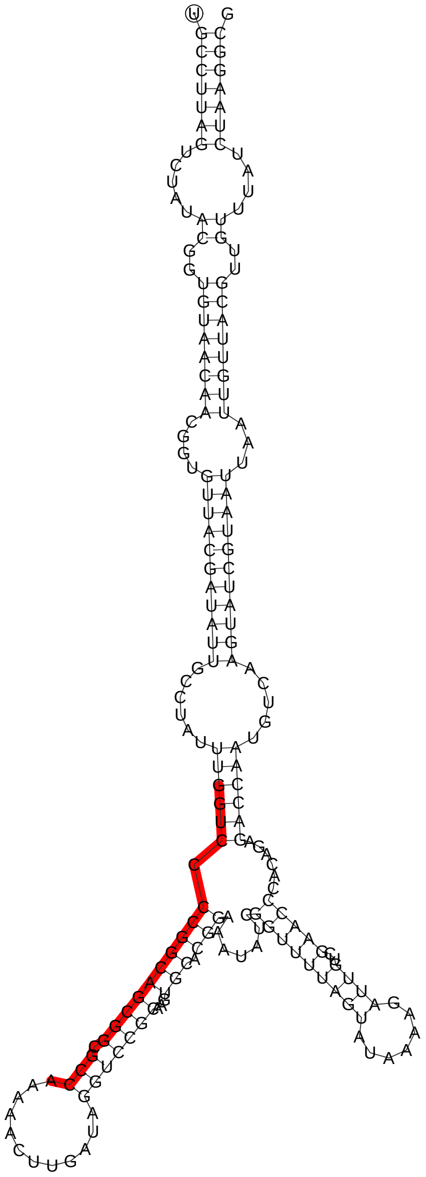

Secondary structure for 'C5'

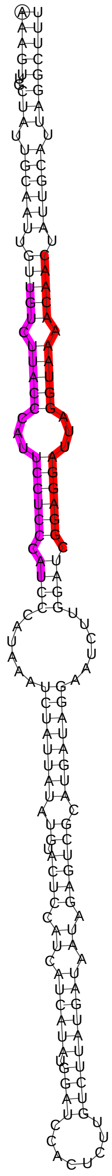

Secondary structure for ’C6’

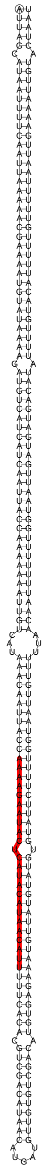

Secondary structure for ’C7’

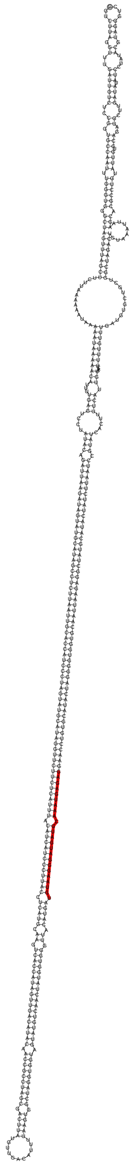

Secondary structure for ’C8’

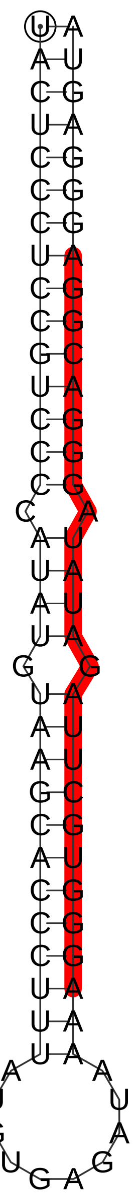

Secondary structure for ’C9_1’

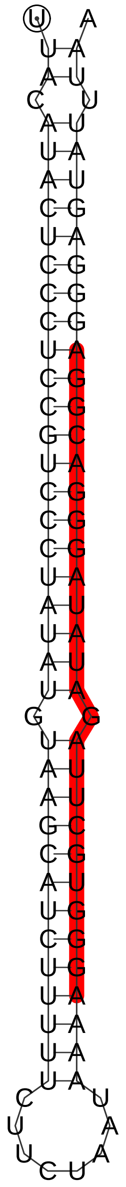

Secondary structure for ’C9_2’

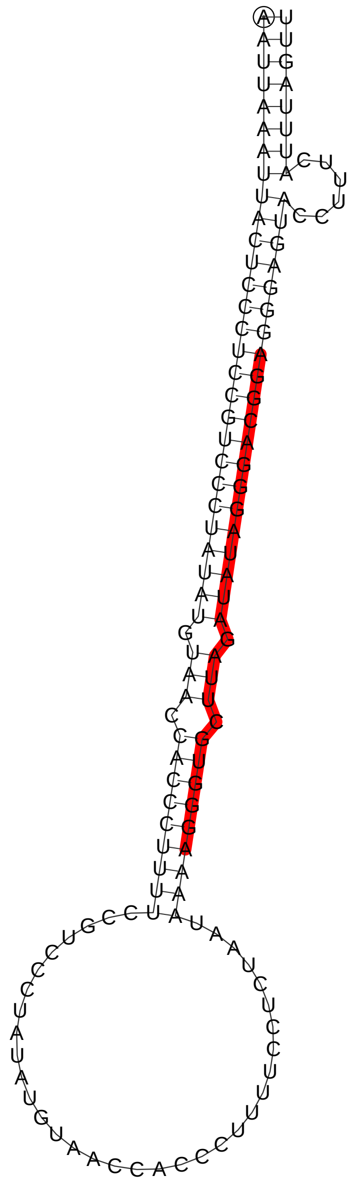

Secondary structure for ’C9_3’
