# Supplementary material for: High-throughput sequencing of Medicago truncatula short RNAs identifies eight new miRNA families
Source: BMC Genomics. 2008 Dec 9;9:593. doi: 10.1186/1471-2164-9-593 (PMC2621214; doi:10.1186/1471-2164-9-593)
Supplement: Additional file 4 — Predicted targets of new validated M. truncatula miRNAs. This table provides a list of predicted target genes of new M. truncatula miRNAs with sequenced miRNA*. [file 1471-2164-9-593-S4.doc]

| Additional File 4. Predicted targets of new validated *M. truncatula* miRNAs | | | |
| --- | --- | --- | --- |
| sRNA ID | target gene accession | start-end position of target | target description |
| miR2086 (1164) | NP7271534 | 51-71 | GB|AC148918.3|ABD28512.1 hypothetical protein |
| miR2086 (1164) | TC125570 | 1402-1421 | homologue to UniRef100_A5AP73 Cluster: Ubiquitin carrier protein; n=1; Vitis vinifera|Rep: Ubiquitin carrier protein - Vitis vinifera (Grape) |
| miR1510b (721) | No target found | N/A | N/A |
| miR1507 (1170) | NP7269413 | 818-838 | GB|AC158497.2|ABN08727.1 Disease resistance protein; Aminoacyl-tRNA synthetase |
| miR1507 (1170) | TC128248 | 826-847 | Disease resistance protein |
| miR1507 (1170) | NP7274631 | 6-24 | GB|AC136955.20|ABE81300.1 Zinc finger |
| miR1510a (170) | No target found | N/A | N/A |
| miR1509 (90) | TC131818 | 189-209 | similar to UniRef100_A0EJF0 Cluster: Beta-glucan-binding protein 1; n=1; Medicago truncatula|Rep: Beta-glucan-binding protein 1 - Medicago truncatula (Barrel medic) |
| miR2087 (35) | No target found | N/A | N/A |
| miR2088 (11) | TC126233 | 2543-2562 | Peptidyl-prolyl cis-trans isomerase |
| miR2089 (9) | NP7254419 | 14-35 | GB|AC153002.15|ABP03463.1 R 4 protein |
| MIR2089 (9) | TC112724 | 650-671 | TIR; Disease resistance protein |
| MIR2089 (9) | NP7254300 | 644-665 | GB|AC156629.26|ABE86370.1 TIR; Disease resistance protein |
| MIR2089 (9) | TC112554 | 645-665 | TIR; Disease resistance protein |
| MIR2089 (9) | NP7254297 | 279-299 | GB|AC156629.26|ABO83726.1 Disease resistance protein; AAA ATPase |
| MIR2089 (9) | TC123065 | 592-613 | weakly similar to UniRef100_Q19PJ2 Cluster: TIR-NBS-LRR type disease resistance protein; n=1; Populus trichocarpa|Rep: TIR-NBS-LRR type disease resistance protein - Populus trichocarpa (Western balsam poplar) (Populus balsamiferasubsp. trichocarpa) |
| MIR2089 (9) | NP7268588 | 437-458 | GB|AC124959.11|ABE83871.2 TIR; Disease resistance protein |
| MIR2089 (9) | DY616742 | 428-449 | weakly similar to UniRef100_Q84ZU5 Cluster: R 8 protein; n=2; Glycine max|Rep: R 8 protein - Glycine max (Soybean) |
| MIR2089 (9) | NP7254391 | 200-221 | GB|AC153002.15|ABE87494.1 Disease resistance protein |
| MIR2089 (9) | NP7258605 | 641-662 | GB|AC127020.16|ABO78819.1 TIR; Disease resistance protein |
| MIR2089 (9) | TC125935 | 656-677 | TIR; Disease resistance protein; AAA ATPase |
| MIR2089 (9) | TC124604 | 733-754 | TIR; Disease resistance protein; AAA ATPase |
| MIR2089 (9) | TC112546 | 638-659 | TIR; Disease resistance protein; Tumor Necrosis Factor |
| MIR2089 (9) | NP7268591 | 635-656 | GB|AC124959.11|ABE83883.2 TIR; Disease resistance protein; KRAB box; Calcium-binding EF-hand; Tumor Necrosis Factor |
| MIR2089 (9) | NP7258611 | 1059-1079 | GB|AC127020.16|ABO78825.1 TIR; Disease resistance protein |
| MIR2089 (9) | NP7267280 | 681-701 | GB|AC144502.5|ABP02775.1 TIR |
| MIR2089 (9) | TC117255 | 676-696 | Disease resistance protein |
| MIR2089 (9) | TC121800 | 722-742 | TIR; Disease resistance protein |
| MIR2089 (9) | TC119737 | 282-302 | TIR |
| MIR2089 (9) | NP7264464 | 282-302 | GB|AC137667.18|ABO79632.1 TIR |
| MIR2089 (9) | TC118553 | 346-364 | weakly similar to UniRef100_Q84VZ7 Cluster: At2g03440; n=1; Arabidopsis thaliana|Rep: At2g03440 - Arabidopsis thaliana (Mouse-ear cress) |
| MIR2089 (9) | TC119536 | 3229-3249 | RNA-binding region RNP-1 (RNA recognition motif) |
| MIR2089 (9) | TC130890 | 683-704 | TIR; Disease resistance protein; AAA ATPase |
| MIR2089 (9) | TC134981 | 679-698 | TIR; Disease resistance protein |
| MIR2089 (9) | AL381638 | 217-237 | weakly similar to UniRef100_Q8WTJ2 Cluster: Dihydropteridine reductase; n=1; Physarum polycephalum|Rep: Dihydropteridine reductase - Physarum polycephalum (Slime mold) |
| MIR2089 (9) | TC125612 | 703-723 | weakly similar to UniRef100_Q19PN7 Cluster: NBS type disease resistance protein; n=1; Populus trichocarpa|Rep: NBS type disease resistance protein - Populus trichocarpa (Western balsam poplar) (Populus balsamiferasubsp. trichocarpa) |
| MIR2089 (9) | TC112592 | 609-629 | TIR |
| MIR2089 (9) | NP7267276 | 681-701 | GB|AC144502.5|ABP02781.1 TIR |
| MIR2089 (9) | NP7276722 | 549-569 | GB|AC149050.2|ABD32877.1 Disease resistance protein; Calcium-binding EF-hand; AAA ATPase |
